# Development of Chitosan Functionalized Magnetic Nanoparticles with Bioactive Compounds

**DOI:** 10.3390/nano10101913

**Published:** 2020-09-25

**Authors:** Gordana Hojnik Podrepšek, Željko Knez, Maja Leitgeb

**Affiliations:** 1 Laboratory for Separation Processes and Product Design, Faculty of Chemistry and Chemical Engineering, University of Maribor, Smetanova ul. 17, 2000 Maribor, Slovenia; gordana.hojnik@um.si (G.H.P.); zeljko.knez@um.si (Ž.K.); 2 Faculty of Medicine, University of Maribor, Taborska ulica 8, 2000 Maribor, Slovenia

**Keywords:** maghemite, metal oxides, magnetization curve, chitosan, enzyme immobilization, cholesterol oxidase, horseradish peroxidase, reusability

## Abstract

In this study, magnetic maghemite nanoparticles, which belong to the group of metal oxides, were functionalized with chitosan, a non-toxic, hydrophilic, biocompatible, biodegradable biopolymer with anti-bacterial effects. This was done using different synthesis methods, and a comparison of the properties of the synthesized chitosan functionalized maghemite nanoparticles was conducted. Characterization was performed using scanning electron microscopy (SEM) and vibrating sample magnetometry (VSM). Characterizations of size distribution were performed using dynamic light scattering (DLS) measurements and laser granulometry. A chitosan functionalization layer was confirmed using potentiometric titration on variously synthesized chitosan functionalized maghemite nanoparticles, which is important for further immobilization of bioactive compounds. Furthermore, after activation of chitosan functionalized maghemite nanoparticles with glutaraldehyde (GA) or pentaethylenehexamine (PEHA), immobilization studies of enzyme cholesterol oxidase (ChOx) and horseradish peroxidase (HRP) were conducted. Factors influencing the immobilization of enzymes, such as type and concentration of activating reagent, mass ratio between carrier and enzyme, immobilization time and enzyme concentration, were investigated. Briefly, microparticles made using the chitosan suspension cross-linking process (MC2) proved to be the most suitable for obtaining the highest activity of immobilized enzyme, and nanoparticles functionalized with chitosan using the covalent binding method (MC3) could compete with MC2 for their applications.

## 1. Introduction

Magnetic metal oxides exhibit exceptional micromagnetic properties, such as magneto-caloric effect, magneto-optic effect and superparamagnetic behavior, which makes them technologically important. Some of the applications of nanocrystalline metal oxides are key components in the production of ceramics, catalysis, sensors, transparent conductive films, electro-optical and electro-chromic devices [1,2]. Metal oxide nanoparticles are also increasingly used in biomedical engineering and in biocatalysis [3]. 

Recently, iron oxide and ferrites such as maghemite (γ-Fe_2_O_3_) have attracted the attention of researchers because of their unique size, physical properties, and their wide applications in different processes as a result of their controllable sizes, shapes and compositions [4]. Furthermore, coating metal oxide magnetic nanoparticles with chemically active polymers such as chitosan can protect them from oxidation and provide amino functional groups for attachment [5,6]. 

Chitosan, a deacetylated derivative of chitin, is a polysaccharide with both hydroxyl and amine groups in its structure, which play an important role in cross-linking with other molecules, such as proteins, making it suitable for various commercial applications [7]. In addition, it is non-toxic, hydrophilic, biocompatible, biodegradable, and anti-bacterial [8,9].

**Figure 1:**
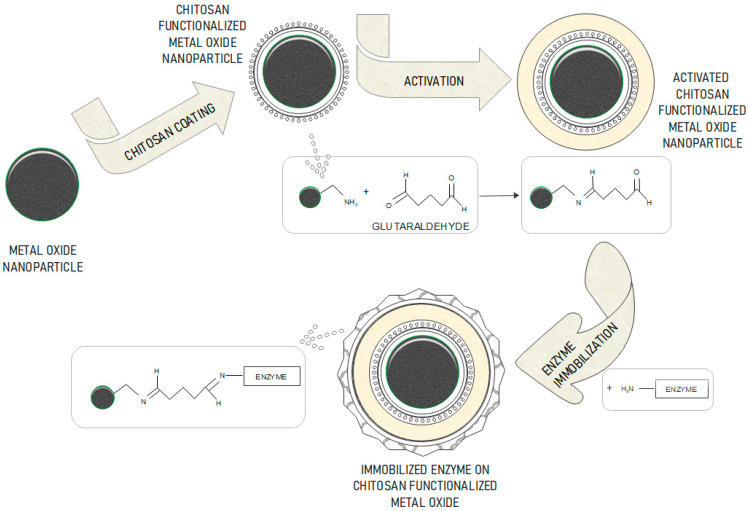
Schematic representation of the entire process of chitosan functionalization, activation and enzyme immobilization on a metal oxide nanoparticle, with chemistry mechanism.

The preparation of chitosan modified magnetic nanoparticles is of great interest to researchers [10,11]. This polymer has been used successfully to colloidally stabilize metal oxide nanoparticles, and it has also been used as a matrix for enzyme immobilization through numerous amino groups, which interact with enzymes as shown in Figure 1 [6]. 

Enzyme immobilization techniques are receiving more interest in enzyme technology, which is currently expanding in various fields such as bioorganic synthesis, biosensors and diagnostics, as well as in industrial processes.

Sahin et al. synthesized chitosan coated magnetic nanoparticles on which glucose-6-phosphatedehydrogenase (G6PD) was immobilized; the optimum pH, temperature and reusability of the immobilized enzyme were studied. According to their results, the immobilized G6PD showed optimum activity at pH 8.0 with a phosphate buffer at 55 °C. After eleven repeated uses, the immobilized G6PD sustained 29% of its initial activity [5]. Laochai et al. synthesized magnetic nanoparticles using a simple co-precipitation method in an aqueous medium incorporated into a chitosan and poly (vinyl alcohol) cryogel microbar and applied them to immobilize the horseradish peroxidase for hydrogen peroxide detection. However, the specific activities of the HRP enzyme were not specified [12]. Some researchers have focused on the preparation of a magnetic carrier as a base through which a layer of chitosan is applied for subsequent immobilization of HRP on a glassy carbon electrode [13], on the surface of a polythionine modified glassy carbon electrode in combination with chitosan and cross-linking of glutaraldehyde [14], and on a Fe_3_O_4_/chitosan modified glassy carbon electrode [15], which was successfully used for the amperometric determination of H_2_O_2_. A recent study by Waifalkar et al. focused on HRP immobilization on a magnetic nanoparticle decorated with graphene, because of its high specific surface area, exceptional electrical conductivity and biocompatibility [16]. 

Several studies have been done on the immobilization of ChOx on chitosan carriers in various forms such as chitosan beads [17], a carbon nanotube-chitosan-platinum matrix [18], and a chitosan-alginic acid network [19]. 

Our work reports on the preparation of chitosan functionalized maghemite nanoparticles. Chitosan’s unique physicochemical and biological properties make it suitable for use in pharmaceutical and biomedical applications [20]. When selecting an appropriate carrier and enzyme immobilization method, several parameters must be considered. The most important are catalytic activity and various stability factors, including thermal, pH, operational and storage. An undesirable phenomenon in the industry is the inconsistency of the rate of release of enzymes from some carriers, which is a very important factor in the immobilization of the enzyme and the proper selection of a carrier. In our study, cholesterol oxidase (ChOx) or horseradish peroxidase (HRP) were immobilized on the surface of differently prepared carriers. The residual activity and immobilization efficiency of these two immobilized enzymes were defined in order to determine the suitability of the carrier. 

HRP is one of the heme peroxidases, which catalyze a variety of oxidative transformations of organic and inorganic substrates with hydrogen peroxide or alkyl peroxides. This is very useful in wastewater remediation, various biotechnological applications and for textile effluent treatment [21,22,23,24].

ChOx catalyzes both oxidation and isomerization of cholesterol to cholest-4-en-3-one. It acts specifically on the 3β-hydroxyl group of cholesterol to produce corresponding ketones using molecular oxygen as an electron acceptor, thus generating hydrogen peroxide (H_2_O_2_). In addition, the enzyme catalyzes the isomerization reaction of cholest-5-en-3-one to cholest-4-en-3-one. Cholesterol oxidase is a bifunctional flavin-adenine-dinucleotide (FAD) dependent enzyme which is widely used to monitor cholesterol levels [25]. It is also important for industrial applications such as bioconversions [26].

To the best of our knowledge, ChOx immobilization on chitosan functionalized magnetic nanoparticles has not yet been explored. Until now, the immobilization of ChOx has been performed on different carriers, such as NiFe_2_O_4_/CuO/FeO-chitosan nanocomposite [27], PS/PEG and PS/PEG/CR particles [28], and a cross-linked matrix of chitosan (Chi)– ionic liquid (IL) (1-butyl-3-methylimidazolium tetrafluoroborate, which was modified with the electrodeposition of Au particles onto thiol (–SH) functionalized multi-walled carbon nanotubes (MWNTs), or shorter versions (MWNT(SH)–Au/Chi–IL/) [29]. In the literature, the activity of the immobilized ChOx is not reported; however, the activity of the biosensor in terms of repeated use has been described. In most cases, retained activity after repeated use, even up to 12 times, is good [17]. Solanki et al. described the immobilization of cholesterol esterase (ChEt) and cholesterol oxidase (ChOx) via glutaraldehyde as a cross-linker onto sol–gel-derived silica (SiO_2_)/chitosan (CHIT)/multi-walled carbon nanotubes (MWCNT)-based nanobiocomposite film deposited onto indium-tin-oxide (ITO) glass. A SiO_2_/CHIT/MWCNT/ITO electrode was used as an immobilization matrix for ChEt and ChOx to fabricate cholesterol biosensors [30]. Šulek et al. reported covalent immobilization of cholesterol oxidase (ChOx) to magnetic nanoparticles of maghemite (γ-Fe_2_O_3_), which was functionalized by silica (SiO_2_) and aminosilane molecules. The activity of the bound enzyme was retained by up to 60% [31]. In our tests, the activity of immobilized ChOx on chitosan functionalized metal oxide nanoparticles was improved to 74.5%. Similarly, Križnik et al., described the preparation of magnetic maghemite nanoparticles functionalized with amino-organosilanes and chitosan for immobilization of the enzyme β-galactosidase from *Aspergillus oryzae*. The highest residual activity of 129% was obtained by using a combination of PEHA and GA (30% and 0.5% (*v*/*v*)) with immobilization on magnetic maghemite nanoparticles functionalized with chitosan [32].

The direct immobilization of HRP and ChOx enzymes on chitosan functionalized metal oxide micro- and nanoparticles for possible use in enzyme-based biosensors reported in this article contributes an important new development in this research field.

## 2. Experimental

### 2.1. Materials

Chitosan (CTS, MMW, degree of deacetylation 75–85%), glutaraldehyde (GA) and Span-80 were obtained from Sigma-Aldrich. Iron (II) chloride tetrahydrate (FeCl_2_·4H_2_O), iron (III) chloride hexahydrate (FeCl_3_·6H_2_O) and acetic acid were supplied by Merck (Darmstadt, Germany). Pentaethylenehexamine (PEHA), dipotassium hydrogen phosphate (K_2_HPO_4_), and potassium dihydrogen phosphate (KH_2_PO_4_) were purchased from Acros Organics (Nidderau, Germany). Ammonia was purchased from Carlo Erba (Val de Reuil, France), and paraffin oil from Pharmachem (Ljubljana, Slovenia). Horseradish peroxidase (HRP, EC 1.11.1.7; 303 U/mg) and Cholesterol oxidase (ChOx, EC 1.1.3.6; 19.8 U/mg) were purchased from BBI Enyzmes (London, UK). All reagents used were of analytical grade without further purification. All solutions were prepared with Milli-Q water. 

### 2.2. Synthesis of Magnetic Maghemite Nanoparticles and Magnetic Fluid

Maghemite nanoparticles were synthesized by hydrothermal coprecipitation of Fe^2+^ and Fe^3+^ ions in the presence of ammonium. First, 2.684 g of FeCl_2_·4H_2_O and 3.11 g of FeCl_3_·6H_2_O were dissolved in 250 mL of double-deionized Milli-Q water and thoroughly mixed at room temperature. The initial pH value was adjusted to 3 (±0.1), with stirring for 30 min. Then, the pH was increased rapidly to 11 (±0.1) by adding 25% of the ammonia solution directly to the solution of iron ions under vigorous stirring. Finally, the γ-Fe_2_O_3_ precipitates were washed with deionized water, collected by magnetic decantation and dried in a vacuum oven at 70 °C [33].

For the preparation of magnetic fluid, the surface of magnetic nanoparticles of maghemite was coated with a surfactant (citric acid) to prevent agglomeration of the particles. The washed magnetic nanoparticles were dispersed in distilled water, to which 2.5 mL of citric acid solution (2.5 g of citric acid dissolved in 5 mL of distilled water) was added. The acid prevents the agglomeration of nanoparticles and thus contributes to the greater stability of the suspension. The pH was adjusted to 5.2 ± 0.1 with 25% ammonia gradually added. At this pH, the adsorption of acid on the nanoparticles is at its most efficient and consequently, the mass fraction of maghemite particles in the magnetic fluid is the largest. 

### 2.3. Synthesis of Chitosan Functionalization

The functionalization of chitosan was carried out using three methods; the microemulsion process (MC1) [34], suspension cross-linking (MC2) [35] and the covalent binding method (MC3) [36]. The optimization of chitosan functionalized magnetic nanoparticles synthesis processes was previously performed, and thus the optimized processes according to the properties of the particles have been used.

#### 2.3.1. Microemulsion Process

For the production of chitosan functionalized maghemite microparticles using the microemulsion process, 0.5 g of chitosan was dissolved in 2% acetic acid solution. Chitosan forms a colloidal solution with acetic acid. Maghemite nanoparticles were added, and the mixture was exposed to an ultrasonic bath for 30 min and stirred several times. 80 mL of paraffin oil and 4 mL of emulsifier (Span-80) were added to the mixture in the water bath at 40 °C. Then, 2 mL of glutaraldehyde (25% (*v*/*v*)) was added slowly to the mixture, which was stirred vigorously at 40 °C for 60 min. The pH of the mixture was adjusted to 9 (with 1M NaOH) and the water bath was heated to 70 °C. After 60 min, the chitosan functionalized microparticles were separated from the viscous mixture using an external magnetic field, washed with ethanol and distilled water, and dried in an oven at 60 °C.

#### 2.3.2. Suspension Cross-Linking Process

Maghemite nanoparticles were dispersed in a mixture of paraffin oil (30 mL) and emulsifier (Span-80; 0.5 mL). Separately, chitosan (0.2 g) was dissolved in 5% acetic acid. Both mixtures were combined and exposed to an ultrasonic bath for 30 min. After the addition of 3 mL of glutaraldehyde (25% (*v*/*v*)), the mixture was stirred at room temperature for 4 h using a mechanical stirrer. Subsequently, chitosan functionalized maghemite microparticles were isolated with an external magnetic field and dried in an oven at 60 °C.

#### 2.3.3. Covalent Binding Method

A solution of chitosan (0.035 g dissolved in 3 mL distilled water) and 7 g of magnetic fluid were dissolved in 3.5 L of distilled water. The pH of the mixture was lowered to 3.7 with 1 M HCl and the mixture was treated in an ultrasonic bath for 60 min at 60 °C. The vessel was equipped with a mechanical stirrer and stirred for 12 h to allow the chitosan to covalently bond to the metal oxide nanoparticles. Then, the mixture was placed on a magnet for 24 h and the chitosan functionalized maghemite nanoparticles were settled. The supernatant was discarded and the particles were washed with distilled water and air dried.

### 2.4. Characterization of Metal Oxide Nanoparticles and Chitosan Functionalized Metal Oxide Micro- and Nanoparticles

#### 2.4.1. Scanning Electron Microscopy (SEM)

The size and morphology of metal oxide nanoparticles and chitosan functionalized metal oxide micro- and nanoparticles were analyzed with scanning electronic microscopy (FE, SEM SIRION, 400 NC, FEI, Oxford Instruments, Abington, UK). The sample for SEM analysis was prepared by placing dried metal oxide nanoparticles or chitosan functionalized metal oxide micro- and nanoparticles onto a copper grid, which was further coated with Au.

#### 2.4.2. Transmission Electronic Microscopy (TEM) with Energy Dispersive X-ray Analysis (EDS)

The structure and size of chitosan functionalized metal oxide micro- and nanoparticles were observed using transmission electron microscopy (HRTEM, JOEL 2010 F, Joel Ltd., Tokyo, Japan). The MC1, MC2 and MC3 samples were prepared in an ethanol/water = 1/1 mixture, in which the particles were diluted 10×. Energy dispersive X-ray analysis (EDS) is a technique used to identify the elemental composition of a sample or a small area of interest on the sample.

#### 2.4.3. Particle Size Analysis 

The particle size distribution of metal oxide nanoparticles and chitosan functionalized metal oxide microparticles were measured with a laser diffraction particle size analyzer (Analysette 22, Fritsch GmbH, Idar-Oberstein, Germany), which operates on the principle of laser diffraction spectrometry with a wet method, and which analyzes particles in the 0.3 to 300 µm range. Particle size distribution is reported both as a Gaussian curve and an arithmetic mean [µm]. Particle size distributions were determined in triplicate.

Dynamic Light Scattering (DLS) was used for particle size determination of metal oxide nanoparticles, where the hydrodynamic diameter of nanoparticles in solution was measured.

#### 2.4.4. Amino Group Determination

In potentiometric titration, a glass electrode, which is an ideal potentiometric sensor for monitoring changes in the concentration of oxonium ions during titration, is used. This process can be detected by changing the pH value. The amino groups of chitosan in the acid medium are protonated into NH^3+^ groups. Protonation of amino groups is important, because the neutralized hydrogen atoms change the potential of the titrated solution. Thus, the molar concentration (Q) of amino groups in solution that are important for further immobilization of enzymes can be indirectly evaluated. pH potentiometric titrations of pure chitosan and chitosan functionalized maghemite micro- and nanoparticles were carried out with a two-burette Mettler Toledo T70 in an inert atmosphere (N_2_ bubbling). The burettes were filled with 0.1 M HCl and 0.1 M KOH. All solutions were prepared in Mili-Q water with low carbonate content (<10^−5^ M). 0.1 g of metal oxide nanoparticles and chitosan functionalized metal oxide micro- and nanoparticles (MC1, MC2 and MC3) was used in the titration process. The suspension was titrated in a back and forth manner between the initial pH = 2.8 to the preset pH = 11. The titration experiments were carried out at 0.1 M ionic strength, set to its appropriate value with KCl. The pH value was measured with a Mettler Toledo DG-117 combined glass electrode. A control HCl-KOH titration was carried out under the same conditions as above [37].

### 2.5. Preparation of GA- and PEHA-Activated Supports

To apply GA activation, an appropriate amount of chitosan metal oxide nanoparticles was functionalized with 4% (*v*/*v*) GA, and the reaction time was estimated at 2 h. The optimum pH value of the reaction medium was 8.0. Chitosan functionalized maghemite micro- and nanoparticles activated with GA were washed with PBS (0.2 M, pH 7.0) to remove any remaining traces of GA.

The surface of the chitosan functionalized metal oxide micro- and nanoparticles was then activated with a monolayer of PEHA, a reagent used to activate the functional groups. Prepared chitosan functionalized maghemite micro- and nanoparticles (following the three different procedures), 1 mL of buffer pH 7.3, and a specific amount of 0.02 M PEHA were mixed and the mixture was shaken at room temperature for a specific time. The mixture was then placed on a magnet to allow the particles to settle to the bottom, and the supernatant was separated from the particles.

### 2.6. Immobilization of HRP and ChOx on Chitosan Functionalized Maghemite Nanoparticles

For HRP immobilization on GA activated, chitosan functionalized metal oxide micro- and nanoparticles (GA-CH-γ-Fe_2_O_3_), PBS buffer and a certain HRP enzyme concentration were added to activated chitosan functionalized metal oxide nanoparticles. This was followed by shaking for 24 h at room temperature. During this time, immobilization of the HRP enzyme on chitosan functionalized magnetic micro- and nanoparticles was performed. Immobilization conditions such as time (3 h, 5 h and 24 h), mass ratio between carrier and enzyme (1:1–1:100) and GA concentration (3.0–8.0% (*v*/*v*)) were optimized. Finally, the resulting chitosan functionalized maghemite micro- and nanoparticles with immobilized HRP were washed with a PBS buffer and a supernatant was used to determine the immobilization efficiency and activity of the immobilized enzyme.

For ChOx immobilization, GA activated, chitosan functionalized metal oxide micro- and nanoparticles (GA-CH-γ-Fe_2_O_3_), and PEHA activated, chitosan functionalized metal oxide micro- and nanoparticles (PEHA-CH-γ-Fe_2_O_3_) were suspended in 0.9 mL of PBS buffer (10 mM, pH 7.3) with 0.1 mL of a given enzyme concentration and mixed well. 24-h immobilization of the enzyme ChOx on chitosan functionalized magnetic micro- and nanoparticles at room temperature and various shaking was performed.

#### 2.6.1. Protein Assay for Immobilization Efficiency

Protein concentrations were determined using the Bradford spectrophotometric method [38]. Measurements were carried out using a UV-Vis spectrophotometer (Varian Cary Probe 50, Agilent technologies, Santa Clara, CA, USA) at a wavelength of 595 nm. The calibration curve was constructed using bovine serum albumin (BSA) as a standard. The resulting protein concentrations are those which are not adsorbed or bound to the carrier. Thus, the immobilization efficiency was recalculated. All protein determination experiments were performed in triplicate. As for the immobilized enzyme, the amount of protein loaded on the MC1, MC2 and MC3 micro- and nanoparticles was evaluated by comparing the difference between the initial and final protein concentration, as well as the washings in the enzyme solutions, which is presented in Equation (1):

(1) $$\varphi =\frac{\left(c_{i}-c_{s}\right)*V_{s}}{m_{c}}$$

where *φ* is concentration of immobilized enzyme [mg enzyme/carrier], *c_i_* and *c_s_* are the initial and final concentrations of protein [mg/mL], *V_s_* is the volume of the total solution [mL] and *m_c_* is mass of the carrier (chitosan functionalized magnetic micro- and nanoparticles) [g].

The immobilization efficiency (IE) can be determined using Equation (2):

(2) $$IE\left[\%\right]=\frac{Total\;protein\;concentration\;of\;immobilized\;enzyme}{Total\;protein\;concentration\;of\;free\;enzyme}*100$$

#### 2.6.2. Enzyme Activity Measurements

Enzyme activities of HRP and ChOx were evaluated by measuring the absorbance of the colorimetric assay using a UV-Vis spectrophotometer.

The residual activity of immobilized HRP was determined by an activity assay based on the oxidation reaction of 4-aminoantipyrine (4-AAP) in the presence of hydrogen peroxide. Phenol, 4-AAP, and H_2_O_2_ were used as color-generating substrates. The reaction rate was determined by measuring an increase in absorbance at 510 nm resulting from the decomposition of hydrogen peroxide. A mixture of 1.5 mL H_2_O_2_ solution (1.7 mM in PBS) and 1.4 mL phenol solution (containing 0.17 M phenol and 2.5 mM 4-aminoantipyrine in distilled water) was prepared and enzyme solution was added to the mixture; the absorbance at 510 nm was monitored for 5 min in the open cuvette following the initiation of the reaction. One unit of specific activity was defined as the consumption of 1 μmol H_2_O_2_ per milligram of enzyme in 1 min at 25 °C at pH 7.0, calculated according to Equation (3):

(3) $$Enzyme\;activity\;\left[\frac{U}{\mathrm{mg}}\right]=\frac{\Delta A/\min}{\left(\varepsilon *m_{e}\right)*V_{s}}$$

where U/mg is the specific *activity* of the HRP *enzyme* [mol·mg^−1^·min^−1^], ∆*A*/min is the slope of the 4-AAP oxidation reaction curve [min^−1^], *ε* is the molar extinction coefficient [6.58 M^−1^·cm^−1^], *m_e_* is the enzyme mass in the sample [mg] and *V_s_* is the volume of the reaction solution [mL].

The activity of free and immobilized ChOx was determined spectrophotometrically in a reaction mixture containing 400 μL of aqueous sodium acetate solution (100 mM, pH 5) and 100 μL of cholesterol solution (0.5% (*w*/*v*)) as a substrate for the ChOx biocatalyst. The reaction mixture was placed on a shaker and heated to 37 °C. A suspension of immobilized enzyme on a chitosan functionalized magnetic nanocarrier was then added directly to the reaction mixture. An assay mixture in which a sodium phosphate buffer (10 mM, pH 7.3) was used instead of the enzyme served as the control. The resulting mixture was shaken on a shaker for 30 min at 37 °C. ChOx catalyzes the oxidation of cholesterol using oxygen as an electron acceptor to form cholest-4-en-3-one and H_2_O_2_ as products. Therefore, after 30 min, 3 mL of 99.8% ethanol was added to the reaction mixture to stop the reaction. The suspension was then centrifuged at 10,000 rpm for 2 min and the liquid supernatant phase was used to determine the enzyme activity at a wavelength of 243 nm on a UV-VIS spectrophotometer.

One enzyme unit (*U/mL_enzyme_*) is defined as the amount of biocatalyst ChOx that converts during the catalytic reaction of 1 μmole of cholesterol into the ketone product 4-cholesterol-3-one per minute at pH 5.0 and 37 °C.

The specific activity of free and immobilized ChOx was calculated using Equation (4):

(4) $$U/mL_{enzyme}=\frac{A_{sample}-A_{blank}*V_{reaction\;mixture}*df}{t_{reaction}*\varepsilon *V_{sample}}$$

where *U/mL_enzyme_* is the specific activity of the ChOx enzyme, *V_reaction mixture_* indicates the volume of the stopped reaction, *df* is the dilution factor, *t_reaction_* defines the time of the assay, *ε* is the millimolar extinction coefficient of 4-cholesten-3-one, and *V_sample_* is the volume of enzyme used.

The residual activity [%] is defined as the ratio between the specific activity of the immobilized enzyme and the specific activity of the free enzyme. The residual activity [%] was determined using Equation (5):

(5) Residual activity [%]=Umg (immobilized enzyme)Umg(free enzyme)∗100

All experiments were carried out in triplicate and the mean values express the average ± standard deviation.

## 3. Results and Discussion

**Figure 2:**
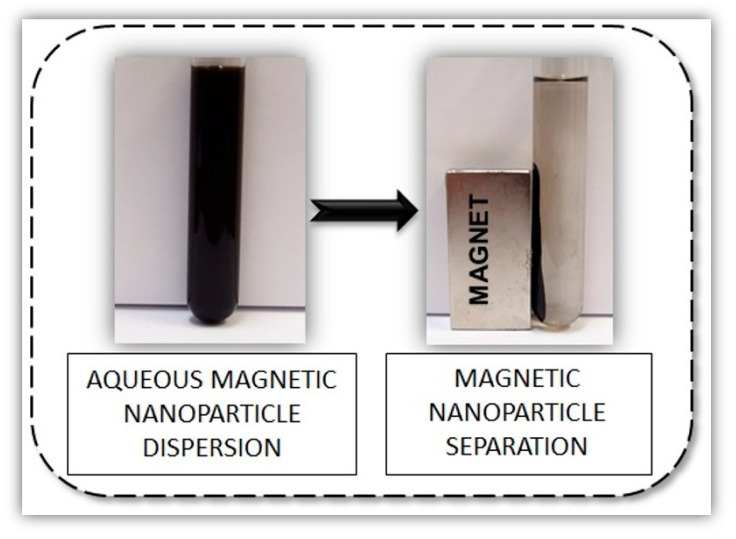
Magnetic properties of maghemite nanoparticles dispersed in water.

Nanoparticles of magnetic metal oxides were synthesized by coprecipitation of ferrous and ferric salts in an alkaline medium. The nature of magnetic nanoparticles and their magnetic properties are significant, since the magnetic properties of metal oxide nanoparticles must remain strong enough even after chitosan coating and enzyme immobilization, which is the focus of this study. Their magnetic response can be manipulated using an external magnetic permanent magnet (2000 Oe), as seen in Figure 2.

GA is a bifunctional agent which was used for the activation of amino groups on chitosan functionalized metal oxide nanoparticles [39]. The mechanism of the reaction between the functional groups of molecules in the carrier preparation process is an important factor in the immobilization process, since free functional groups are required for immobilization of the enzyme. To prepare the dispersion of nanoparticles in a polar solvent, specifically in water, the synthesized metal oxide nanoparticles were coated with citric acid. Since chitosan has in its molecular structure free –NH_2_ and -OH groups, the reaction can be carried out with the carboxylic group of citric acid in two ways. If the reaction is performed between the -OH group of chitosan, an ester is formed which can further react with the amino group of PEHA, which was used to activate the metal oxide nanoparticles before the immobilization process.

A reaction between the carboxylic group of citric acid on the surface of metal oxide nanoparticles and the –NH_2_ group of chitosan is also possible. This produces an amide which can further react with the amino group of PEHA and the aldehyde group of GA and is important as a surface preparation for enzyme immobilization. 

### 3.1. Characterization of Metal Oxide Nanoparticles and Chitosan Functionalized Metal Oxide Micro- and Nanoparticles 

Characterization of metal oxide nanoparticles and chitosan functionalized metal oxide micro- and nanoparticles is important in determining the properties of synthesized particles. In addition, determining the specific characteristic properties of metal oxide allows them to be used in a variety of applications. 

#### 3.1.1. Particle Size Analysis, SEM Analysis and TEM Analysis

The determination of particle size distribution is an important parameter for controlling the synthesis process of metal oxide nanoparticles. During the chitosan coating process, layers of different thicknesses are formed. We determined the effects of chitosan coatings on the magnetic material’s properties and selected the chitosan coating process which allows the highest activity of the immobilized enzyme.

**Figure 3:**
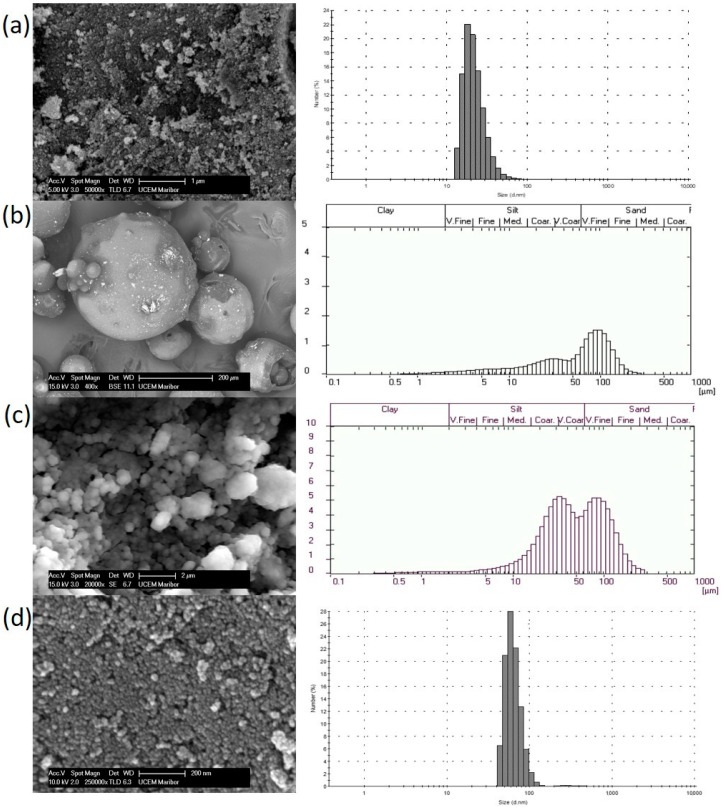
SEM micrographs and particle size distribution of (**a**) bare maghemite, (**b**) magnetic nanoparticles functionalized with chitosan using a microemulsion process (MC1), (**c**) magnetic nanoparticles functionalized with chitosan using a suspension cross-linking process (MC2) and (**d**) magnetic nanoparticles functionalized with chitosan using a covalent binding method (MC3).

Laser diffraction granulometry was used to study the distribution of particle sizes present in samples of chitosan functionalized maghemite using two different synthesis methods. The particle size and particle size distribution of chitosan functionalized maghemite microparticles, MC1 and MC2, were measured with laser granulometry, which operates on the principle of laser diffraction spectrometry using a wet method and analyzes particles in the size range of 0.3–300 µm, as presented in Figure 3. Since this method does not allow measurement of particles smaller than 0.3 µm, dynamic light scattering (DLS) was used to measure the size of bare maghemite nanoparticles and maghemite nanoparticles functionalized with chitosan using the MC3 covalent binding method in the nano sized range. Scanning electron microscopy (SEM) was used to obtain the morphological properties of prepared nanoparticles. A comparison between SEM images and particle size distribution is shown in Figure 3, where it can be seen that bare maghemite is nanometer-sized material and has the lowest size distribution. During the chitosan coating process, particle size increases, as the size of the individual particle is related to the thickness of the chitosan layer.

SEM and DLS analysis (Figure 3a) show that bare metal oxide nanoparticles form aggregates of nearly spherical shaped nanoparticles with a mean diameter of 22.7 nm. SEM micrographs of chitosan functionalized metal oxide micro- and nanoparticles show an increase in particle sizes caused by chitosan coating. The MC1 samples are spherical with a thicker chitosan coating, which is clearly seen in the SEM micrographs in Figure 3b. These metal oxide microparticles, which were functionalized with chitosan using a microemulsion process, have an average size of 5–350 µm and a mean diameter of 68.5 µm. The laser granulometry results of chitosan maghemite microparticles functionalized by the MC2 suspension cross-linking process are shown in Figure 3c, with a mean diameter of 44.2 µm and size distribution from 10 to 200 µm, which is consistent with the measurements of SEM analysis. The mean diameter of magnetic nanoparticles functionalized with chitosan using the MC3 covalent binding method was 58.8 nm, with a range from 50–100 nm (Figure 3d). The maghemite chitosan nanoparticles obtained differed in size and quantity of the functionalized chitosan. In research published by Díaz-Hernández et al., the average size of chitosan functionalized nanoparticles increased slightly after chitosan coating, a finding which is similar to our observations. Their research describes chitosan functionalized particles with a size distribution of 120–300 nm and an average diameter of approximately 230 nm, which were synthesized in a single step alkaline precipitation. In comparison to our results, their study reports magnetite (Fe_3_O_4_) agglomerates with individual spherical particles and an average diameter of 8.5 nm [40]. 

**Figure 4:**
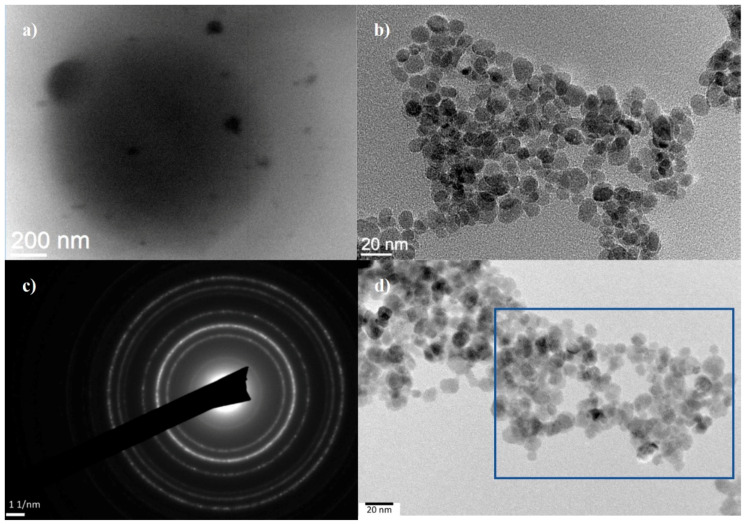
TEM images of (**a**) chitosan functionalized metal oxide microparticles using the microemulsion process (MC1), (**b**) chitosan functionalized metal oxide microparticles using the suspension cross-linking process (MC2), (**c**) the crystallinity of the nanoparticles and (**d**) chitosan functionalized metal oxide nanoparticles using the covalent binding method.

With TEM analysis, it was difficult to evaluate the thin organic coatings on the surfaces of inorganic nanoparticles in the MC1 sample. Figure 4 shows a TEM image of chitosan functionalized metal oxide micro- and nanoparticles using the (a) microemulsion process (MC1), (b) suspension cross-linking process (MC2) and (d) covalent binding method (MC3). The MC1 pattern changed visibly under the electron beam because of the presence of larger amounts of organic material. Larger structures (micron-sized and even larger), which are mostly round in shape and probably composed of chitosan, can be observed in the sample. In this sample, energy dispersive X-ray analysis (EDS) did not show the presence of iron. At the edges of this organic material, however, smaller darker areas representing magnetic nanoparticles can be observed. Moreover, EDS analysis showed the presence of iron in this area. The MC2 microparticles are crystalline, similar to the MC3 sample. At lower magnifications, larger amorphous regions in which metal oxide nanoparticles are trapped can be observed. In the images of TEM analysis, a thin amorphous layer of chitosan can be observed, which most likely encompasses several metal oxide nanoparticles in the cluster. In the MC3 sample, the microparticles are relatively well separated, indicating the presence of chitosan coatings that provide steric stabilization to the particles. Figure 4c records the diffraction, showing the crystallinity of the nanoparticles. This is the area marked with a blue square in Figure 4d.

#### 3.1.2. Magnetic Properties of Chitosan Functionalized and Bare Metal Oxide

**Figure 5:**
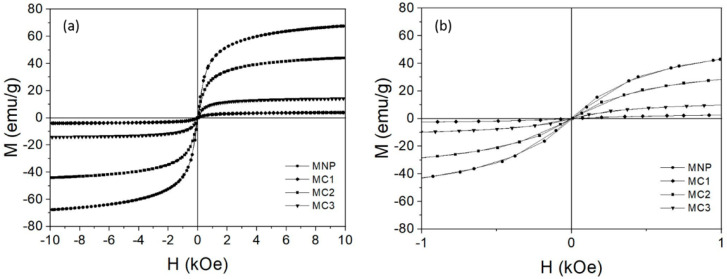
Magnetization curves of chitosan functionalized magnetic nanoparticles in comparison with bare magnetic nanoparticles; the filled circles represent magnetic maghemite nanoparticles (MNP), the filled inverted squares represent magnetic microparticles functionalized with chitosan using the microemulsion process (MC1), the filled squares represent magnetic microparticles functionalized with chitosan using the suspension cross-linking process (MC2), and the filled inverted triangles represent magnetic nanoparticles functionalized with chitosan using the covalent binding method (MC3), where (**a**) presents magnetization curves ranging from −10 kOe to +10 kOe and (**b**) presents magnetization curves near zero magnetic field ranging from −1 kOe to +1 kOe.

To examine the magnetic properties of prepared magnetic oxide core nanoparticles, a vibration magnetometer (VSM) was used. The magnetization curves of maghemite, maghemite nanoparticles functionalized with chitosan using the microemulsion process (MC1), the suspension cross-linking process (MC2) and the covalent binding method (MC3), which are depicted in Figure 5, are typical of superparamagnetic nanoparticles because the remanence and coercivity of the loop are zero and no hysteresis curves are visible [41]. At the same time, the process of magnetization and demagnetization of particles describes the same curve and the magnetizations in both high and low magnetic fields of 10 kOe and 1 kOe are shown in Figure 4a,b, respectively. However, there is no obvious tendency of magnetization deterioration observed for the low magnetic field. 

The saturation magnetization (Ms) value of maghemite nanoparticles in a high magnetic field was approximately 67.5 emu/g. The Ms of chitosan functionalized magnetic microparticles by the suspension cross-linking process (MC2) was 44.1 emu/g; this is significantly lower than their bulk value. The Ms of magnetic nanoparticles functionalized with chitosan using the process of covalent binding (MC3 g) was 14.2 emu/g, and chitosan functionalized magnetic microparticles using the microemulsion (MC1) process was 4.0 emu/g. The value of saturation magnetization depends on the thickness of the chitosan layer, which in turn depends on different processes of chitosan coating around the magnetic core [42]. The chitosan thicknesses can vary depending on the preparation method according to TEM results (Table 1), but they can also vary depending on numerous parameters that exist during each preparation method, such as temperature or processing times. The highest saturation magnetization (Ms) value can also result from a non-uniform coating of chitosan that can expose core metal oxide to the outer atmosphere and eventually lead to poor stability. In general, the preparation method of chitosan functionalization of metal oxide leads to the formation of two different structures: magnetic core–chitosan shell or magnetic multi-cores homogeneously dispersed in chitosan [43]. Lower saturation magnetization of MC1 microparticles could be explained with TEM analysis, where the structure of magnetic multi-cores homogeneously dispersed in chitosan is clearly visible; otherwise, a different magnetic core-chitosan shell structure is observed in MC2 microparticles, leading to higher saturation magnetization of the microparticles. 

In general, these results are consistent with results published by Khmara et al. and Fu et al., who describe a similar effect of reduced Ms in chitosan functionalized magnetic nanoparticles [44,45]. Gregorio-Jauregui et al. obtained values of magnetic saturation of chitosan-coated magnetic nanoparticles (CMNP) of around 65.6 ± 0.1 emu/g [46]. In accordance with these observations, MC2 is the most suitable carrier, as it offers appropriate magnetic properties for applications where magnetic response is significant.

#### 3.1.3. Determination of the Number of Available Amino Groups

In general, potentiometric and conductometric titrations are widely used to quantify the amino groups of chitosan in solution and to determine the degree of deacetylation of chitosan. Potentiometric titration in aqueous solutions is a simple and effective method which is considered to be the most precise method for the determination of protonated amino groups on the surface of pure chitosan and chitosan functionalized magnetic nanoparticles [47].

**Figure 6:**
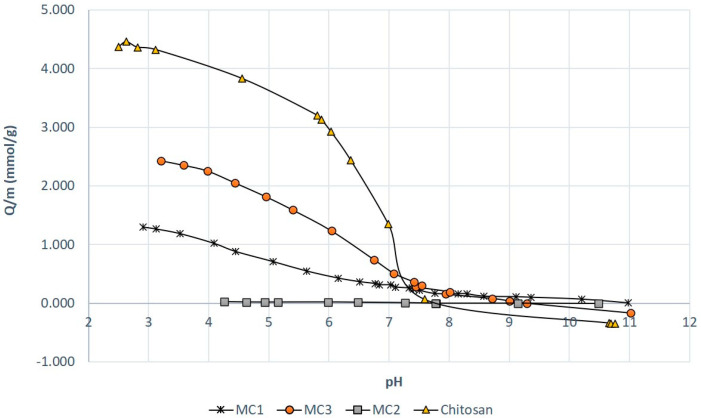
Potentiometric determination of amino groups in magnetic microparticles functionalized with chitosan using the microemulsion process (MC1), magnetic microparticles functionalized with chitosan using the suspension cross-linking process (MC2) and magnetic nanoparticles functionalized with chitosan using the covalent binding method (MC3).

Determination of the number of available amino groups of chitosan functionalized magnetic micro- and nanoparticles by three different methods was performed using potentiometric titration in an aqueous medium, in which the aqueous acid solution was titrated with the base. The total number of weak acidic groups was calculated from the difference (ΔV) in the added KOH volume between the chitosan sample and the control, at any given pH. The number of protonated amino groups as weak acids, as represented in samples of pure chitosan and chitosan functionalized maghemite micro- and nanoparticles, is shown in Figure 6, and was calculated from the potentiometric titration curves.

Figure 6 shows that pure chitosan has, as expected, the highest molar concentration of available amino groups, 4.22 mmol/g. After functionalization of magnetic nanoparticles with chitosan, the molar concentration of amino groups decreased. The highest molar concentration of available amino groups was obtained for chitosan functionalized magnetic nanoparticles by the covalent bonding method (MC3), which is 2.48 mmol/g. It has been reported that the concentration of free amino groups of chitosan on the surface of magnetic nanoparticles depends on the method of binding of chitosan to the magnetic carrier and the orientation of the chitosan molecule [48]. Nevertheless, magnetic microparticles functionalized with chitosan using the microemulsion method (MC1) contained 1.18 mmol/g of available amino groups, and magnetic microparticles functionalized with chitosan using the suspension cross-linking process (MC2) only 0.02 mmol/g. The positive charge on the surface of magnetic nanoparticles is caused by the adsorption of chitosan on magnetic nanoparticles and the availability of its amine groups. We conclude that the highest binding affinity is found for chitosan functionalized magnetic nanoparticles by the covalent binding method (MC3) and the lowest for chitosan functionalized magnetic microparticles by the suspension cross-linking process (MC2). The reasons can be attributed to physico-chemical properties such as presence of amino groups, the degree of protonation of NH_2_ [49], and especially the structural properties of the carrier. The method of functionalization with chitosan on the surface of magnetic nanoparticles plays an important role as well. Physical interactions including electrostatic, hydrophobic and affinity interactions are especially important in the binding of molecules to nano-sized carriers. 

### 3.2. Chitosan Functionalized Metal Oxide Nanoparticles for Enzyme Immobilization

There are many potential applications in biomedicine for chitosan functionalized metal oxide nanoparticles, such as drug delivery systems [50], magnetic resonance imaging (MRI) [51] and magnetic hyperthermia [52]. We look forward to additional advances in the use of chitosan functionalized metal oxide micro- and nanoparticles as carriers for enzyme immobilization.

#### 3.2.1. Immobilization of HRP and ChOx Using GA as an Activation Reagent

Immobilization of soluble enzymes to a suitable support matrix is the most effective means of optimizing specific properties of the enzymes (catalytic activity, stability and specificity) [53]. Moreover, the use of immobilization technology for biocatalysts is increasingly significant for industry, and it is therefore very important from an economic point of view. HRP and ChOx immobilization on chitosan functionalized metal oxide micro- and nanoparticles can improve operational stability and thus provide a basis for obtaining enzymes from a reaction medium that allows continuous operation with reuse of the immobilized enzymes. 

Optimization of HRP immobilization was performed in our study by varying the mass ratio between carrier and enzyme, concentration of glutaraldehyde and immobilization time. Also, a comparison of the immobilization efficiency and residual activity of enzymes immobilized onto chitosan functionalized metal oxide micro- and nanoparticles was performed. Covalent cross-linking of polymer chains is used to increase matrix density and mechanical strength. Furthermore, GA and PEHA as activating reagents exhibit good mechanical properties. Thus, a higher immobilization yield can be expected as a result of the presence of a larger number of reactive groups. The use of these agents allows modulation of some structural properties, hydrophobic behavior and the amount and activity of immobilized target enzymes of chitosan matrixes [54]. 

The immobilization process was optimized by changing conditions such as time (3 h, 5 h and 24 h), mass ratio between carrier and enzyme (1:1–1:100) and GA concentration (3.0–8.0% (*v*/*v*)), to obtain the highest possible efficiency. Under the initial conditions (GA 3.2%; 24 h immobilization time; mass ratio between carrier and enzyme (1:1)), only 12.8% immobilization efficiency, with very low activity of immobilized HRP (only 0.03% of the calculated specific activity of the free enzyme), was achieved. To improve the result, the weight ratio of carrier to enzyme was gradually increased from 1 to 100. The ratio can be increased either by decreasing the weight of the enzyme or by increasing the weight of the carrier in 1 mL of sample, resulting in a larger active surface for enzyme immobilization.

**Figure 7:**
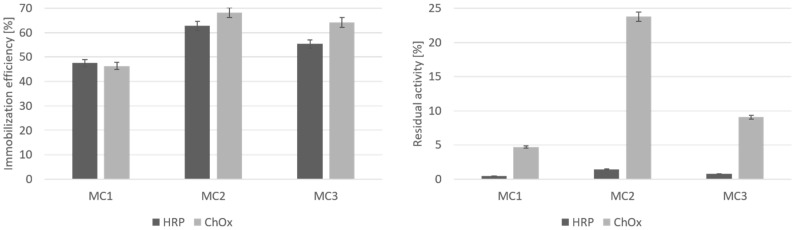
Immobilization efficiency and residual activity of HRP and ChOx on different chitosan functionalized metal oxide micro- and nanoparticles with GA activation.

The immobilization efficiency of the enzyme increases with increasing weight ratio between the carrier and the HRP enzyme. The highest immobilization efficiency of 75.3% was achieved at a weight ratio of 1:100, although under these conditions only 0.7% of the activity of the immobilized HRP enzyme was preserved. In the next step, GA concentration was increased, to enlarge the binding area of the chitosan functionalized magnetic oxide carrier. It is very important, however, not to use too high a GA concentration. Extensive GA concentration may lead to lower enzyme activity, as a result of distortion of the enzyme structure (i.e., the active site conformation) and consequently affecting the retention of enzymatic activity [55]. Figure 7 shows the results of immobilization efficiency and residual activity of immobilized enzymes HRP and ChOx on different supports: chitosan functionalized metal oxide micro- and nanoparticles by the microemulsion process (MC1), suspension cross-linking process (MC2) and covalent binding method (MC3). 

The highest residual activity of immobilized HRP, 0.8%, was obtained using MC2 with 4% GA. The effect of immobilization time was also evaluated because of its direct influence on HRP loading and the possibility of enzyme conformation and denaturation upon immobilization [56]. The activity of the immobilized HRP enzyme increased during the shorter immobilization time. Thus, after 12 h of immobilization, 1.2% of the activity of an MC2 immobilized HRP enzyme was preserved; after 6 h, it was approximately 1.3%, and after 5 h, 1.5%. The highest immobilization efficiency of HRP enzyme, 62.8%, was obtained on chitosan functionalized metal oxide microparticles using the suspension cross-linking process (MC2), which is presented in Figure 6. The optimum HRP enzyme immobilization efficiency was achieved at pH 7, with initial enzyme concentration of 1 mg/mL and immobilization time of 5 h. 

For immobilization of ChOx, first, the activation reagent GA in the concentration range of 1–3% (*v*/*v*) was used to determine the effect of GA concentration on the immobilization efficiency and residual activity of the immobilized ChOx enzyme. The immobilization was performed for 24 h at 300 rpm of shaking with an enzyme concentration of 0.1 mg/mL and 1% (*v*/*v*) of GA. When a higher concentration of GA was used, lower immobilization efficiency and residual activity of immobilized ChOx values were obtained. The immobilization efficiency of ChOx was much higher than the immobilization efficiency of HRP enzyme. The highest obtained immobilization efficiency of ChOx was 68.2% with MC2 and 64.1% with MC3 support, which is presented in Figure 7. It is clear that the residual activity of immobilized ChOx was higher, compared to HRP’s residual activity. The highest residual activity, of up to 23.7%, was obtained from MC2 particles. Chitosan functionalized metal oxide microparticles, synthesized by the suspension cross-linking process (MC2), were found to be the most suitable for the immobilization of enzymes, since both HRP and ChOx provided the highest immobilization efficiency and residual activity of the immobilized enzyme.

It is interesting to note that several studies have reported HRP immobilization in combination with chitosan where a different carrier is mentioned. Luo et al. reported immobilization of HRP on chitosan-wrapped NiFe_2_O_4_ nanoparticles on a glassy carbon electrode (GCE) for practical applications in the development of immunosensors, biosensors and bioelectronic devices [57]. Studies by Bindhu et al. [58], Cao et al. [59] and Mohamed et al. [60] demonstrated the immobilization of HRP on chitosan where the activity retention of the enzyme was investigated. In a study by Cao et al., a different immobilization strategy, a two step ionic gelation method, was used to obtain HRP encapsulated chitosan nanoparticles with 42% maintained activity of free HRP [59]; this study differs from ours in that a particle formation procedure manipulating ionic interaction between positively charged chitosan and negatively charged triphosphate was used. In general, the formation of a bond between chitosan and proteins occurs mainly by electrostatic interactions, a result of the cationic properties of chitosan with proteins such as HRP, which is positively charged [61]. According to the results obtained by Mohamed et al., a maximum immobilization efficiency (60%) was detected at a concentration of 6% cyanuric chloride and pH 5.5, and twelve percent of the initial activity was detected in the washing buffer. In this regard, they also found that the aquaphilicity of the support played an important role in the kinetics of the immobilized HRP in water-miscible solvents. A solvent-aqueous mixture containing the substrate extends to the surface of the biocatalyst because of the hydrophilic nature of the support and this results in high catalytic turnovers. When the concentration of water is low, the amount of available water may not be sufficient to form the aqueous microphase, and this leads to significant changes in the hydration state of the immobilized enzyme, which strongly affects the catalytic activity [58]. 

It is important to note, however, that the binding of enzymes to a magnetic carrier functionalized with chitosan, as described in our study, definitely results in better operational stability and the possibility of multiple use, so that it is also suitable for applications other than just biosensors. 

#### 3.2.2. Immobilization of ChOX Using PEHA as Activation Reagent

There are several important parameters which should be monitored when selecting the appropriate carrier and enzyme immobilization method. The choice of a suitable carrier is an important factor which is evident from the results in the previous section. The other important factors are catalytic activity and thermal, pH, operational and storage stability, and the rate of enzyme release from the carrier; inappropriate release rates are an undesirable phenomenon in industrial applications. In summary, the ideal immobilized biocatalyst for commercial use should possess the following properties: permanent binding to the vehicle, non-toxicity, sufficient resistance to physico-chemical factors in the microenvironment, high catalytic activity and reusability or the possibility to use in a continuous mode [62]. According to the results obtained, our work focused on ChOX because of its low HRP activity, since we wanted to improve the binding and the activity of the immobilized enzyme.

**Figure 8:**
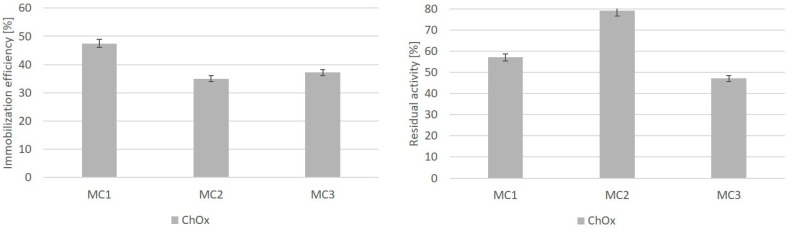
Immobilization efficiency and residual activity of ChOx on different chitosan functionalized metal oxide micro- and nanoparticles with PEHA activation. Each column represents the mean of triplicate values, with the range indicated with error bars.

In the next step, the immobilization of ChOx, PEHA was used as the activation reagent for the formation of a bond between the carrier and enzyme via an epoxy group. Epoxy-activated supports enable very easy protocols for enzyme immobilization. Furthermore, epoxy groups are very stable at neutral pH values and they are able to react with different nucleophilic groups on the protein surface (e.g., amino, hydroxy or thiol moieties) to form extremely strong bonds with minimal chemical modification of the protein [63]. The immobilization process was optimized by changing the enzyme concentration to obtain the highest possible immobilization efficiency. Initially, 0.1 mg/mL and 1.0 mg/mL of ChOx enzyme was used to perform immobilization on chitosan functionalized metal oxide nanoparticles prepared using three different synthesis methods. Briefly, the optimal PEHA concentration used for the activation of chitosan functionalized metal oxide nanoparticles was 0.02 M. Figure 8 shows that the immobilization efficiency of ChOx was the highest (47.5%), using chitosan metal oxide microparticles functionalized by the microemulsion process (MC1) as a carrier. Surprisingly, the activity of immobilized ChOx (79.0%) was the highest, where ChOx was immobilized on chitosan metal oxide microparticles functionalized by the suspension cross-linking process (MC2), which is shown in Figure 8. We assume that the enzyme molecules bind with two different binding sites with higher and lower affinities [64]. Therefore, because of the suitable conformational state of enzyme immobilized on MC2 microparticles and the interaction mechanism between carrier and enzyme, the catalytic activity of the immobilized enzyme was high. Immobilization on a support can increase or decrease the activity of an immobilized enzyme. Moreover, crowding on the surface of a support has been shown to reduce enzymatic activity because of limited active site accessibility, spatial restrictions and denaturation of the protein [65]. Thus, the concentration of the enzyme and binding method is relevant in order to obtain higher activity of the immobilized enzyme. An excessive amount of immobilized enzyme leads to intermolecular steric constraints, which reduce the enzyme activity [66]. MC2 microparticles which were prepared using the chitosan suspension cross-linking process were again confirmed to be the most appropriate for use as a carrier for the immobilization of HRP and ChOx enzymes.

Comparing the results where GA or PEHA were used as activating reagents, the immobilization efficiency of ChOx immobilization was lower when PEHA was used. On the other hand, the residual activity of immobilized ChOx using PEHA was much higher, reaching almost 80% on MC2 support. Previous studies have shown that ChOx activity after immobilization on chitosan beads remained at approximately 20%, compared to free enzyme ChOx at pH value 7.0–8.5 and 50 °C [17].

One of the main problems with enzyme immobilization is the loss of enzyme activity. Usually, for most enzymes, activities are immobilized in a lower form than the activity of the enzymes in their free form. Even though the activity of immobilized HRP enzyme was very low, the immobilization efficiency, which reached a value of 75.3%, proves that the immobilization was successfully performed. The immobilization of the ChOx enzyme was successfully performed with high residual activity up to 79.0%.

#### 3.2.3. Stability of Immobilized ChOX

In general, the stability of the immobilized enzyme and its reusability are important features in the design of various carriers for the immobilization of enzymes for specific purposes and can effectively reduce the cost of its applications. Gradual deactivation and leakage of the enzyme from the system are the most common problems, which strongly encourage the design of immobilized enzymes for their reuse in the process [67]. The stability of immobilized ChOx was determined by reusing the immobilized ChOx on chitosan functionalized magnetic metal oxide micro- and nanoparticles (MC1, MC2, MC3) in a cholesterol oxidation reaction. After each activity measurement, immobilized ChOx enzyme was separated from the bulk magnetically, washed thoroughly with sodium phosphate buffer (10 mM, pH 7.3) and reused in a new cholesterol oxidation reaction until the immobilized ChOx enzyme activity decreased or approached 0. Moreover, to assess the reusability of immobilized ChOx systems, oxidation yields were examined during 9 cycles, in which each cycle lasted for one hour.

**Figure 9:**
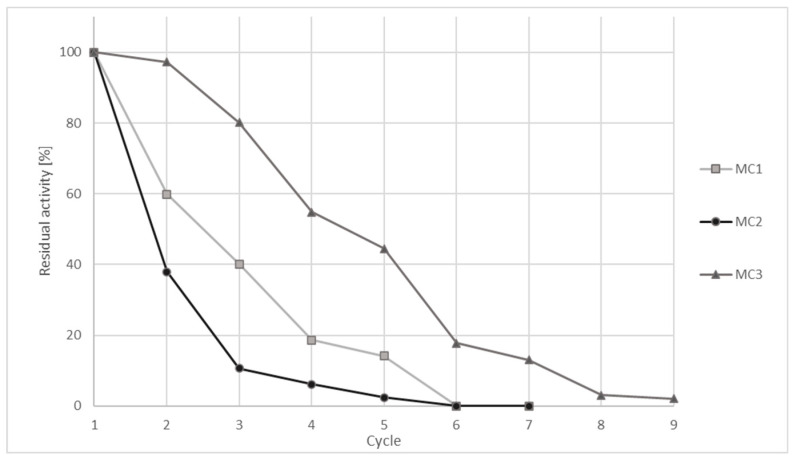
Effect of repeated use of immobilized ChOx on different chitosan functionalized metal oxide nanoparticles (MC1, MC2 and MC3) on enzyme activity.

Figure 9 reveals the effect of repeated use of the immobilized enzyme ChOx on the residual enzyme activity in %. The enzyme activity obtained in the first cycle was considered to be 100% activity. It can be seen from Figure 8 that the residual ChOx activity on MC3 activated with PEHA after 2 cycles was still extremely high, 97.3%. Additionally, immobilized ChOx on MC3 was reused for 5 successive cycles retaining 50% of its initial activity, while it decreased significantly after 9 cycles (2.1%). On the other hand, the residual activity of the immobilized enzyme ChOx on MC1 and MC2 decreased faster. After the second cycle, immobilized ChOx on MC1 possessed 60% of its activity and after 6 cycles, no activity was detected. The stability of immobilized ChOx on an MC2 support was the weakest, since it rapidly decreased below 40% of the initial activity after only 2 cycles of repeated use. 

A similar study on immobilization of ChOx on magnetic fluorescent nanoparticles activated with APTES reports reusability for 7 consecutive operations [68]. Ghosh et al. reported on immobilization of ChOx on silane modified iron (II, III) oxide magnetic nanoparticles, where the residual activity of the nanobioconjugates remained at 50% after 10 cycles [69]. Loss of activity was attributed to the inactivation of the enzymes during repeated recycling, although the immobilization process was developed to improve the stability of the enzyme. The reusability of the immobilized enzyme is a key factor for practical applications in order to reduce costs and simplify processes [70,71].

By using MC2 as a carrier for ChOx immobilization, the best results in terms of ChOX activity and immobilization efficiency were obtained; nevertheless, the use of MC3 nanoparticles allows better stability of immobilized ChOX. 

## 4. Outcome of the Different Coating Methods

In general, functionalization of metal oxide nanoparticles with organic polymers can improve their physical, chemical and biological properties [72]. In addition, chitosan coatings with surface modification could influence further immobilization of enzymes. Table 2 presents the characterization properties of the best-suited chitosan functionalized metal oxide nanoparticles synthesized in this study, where the most important properties are highlighted and the connection with the results obtained in the enzyme immobilization is made. The suspension cross-linking process was successfully used for the synthesis of chitosan functionalized metal oxide microparticles (MC2), where after immobilization, the highest enzyme activity was obtained (79.0%). Also, MC2 microparticles have larger particle size diameters than MC3 nanoparticles and much higher saturation magnetization; the magnetic properties of nanoparticles are important in applications where an external magnetic field is used [73]. However, the activity of immobilized ChOx on MC3 nanoparticles is lower. In addition, there is not a big difference in immobilization efficiency for ChOX when using MC2 (35.0%) or MC3 (37.2%), but a considerable difference in the activity of immobilized ChOx was noticed when using MC2 (79.0%), compared to MC3 (47.1%). 

More available amino groups were associated with better enzyme immobilization efficiency and more stable immobilization, which was demonstrated in MC3 nanoparticles, where we achieved 37.2% immobilization efficiency and 7× reusability of the immobilized enzyme. Also, MC2 microparticles show a lower value of available amino groups, but we show that with the addition of GA or PEHA, immobilization of the enzyme ChOx with 79% residual activity was successfully achieved. The orientation of the amino groups turned out to be different in this case, despite the sufficient thickness of the chitosan coating. As can be seen from TEM analysis, the efficiency of immobilization on MC2 microparticles is not negligible. Thus, it is not only the high activity of the immobilized enzyme that is important, but also the stability of the enzyme on a carrier. Moreover, MC3 produced several amino groups which contribute to the highest immobilization efficiency of 37.2% and improved binding, which is reflected in more successful reusability of immobilized ChOx. Therefore, we find that MC3 could compete with MC2 for any applications.

Procedures optimization of chitosan functionalization on metal oxide nanoparticles by three different methods, was performed in previous studies, therefore, optimized procedures were used in our study according to the properties of the micro- and nanoparticles. For the synthesis of MC1 microparticles, the authors used a different weight ratio of γ-Fe_2_O_3_ nanoparticles to chitosan, where after the synthesis of chitosan functionalized magnetic nanoparticles with a ratio of 2:5, saturated magnetization reached 4.36 emu/g [34]. Accordingly, higher saturation magnetization, MC1 was performed by the same ratio, where similar results were obtained (4 emu/g). Similarly, in the second process of synthesis of magnetic nanoparticles functionalized with chitosan by the suspension cross-linking process, the molar ratio of magnetic nanoparticles to chitosan was optimized. However, MC2 synthesized by the same procedure with a molar ratio 1:1 of magnetic nanoparticles to chitosan, showed higher saturation magnetization, 44.1 emu/g. It is possible, that some of negatively charged magnetic nanoparticles, which were loaded on the surface of microspheres, led to increase of the saturation magnetization [35]. Magnetic nanoparticles functionalized with chitosan by the covalent binding procedure, chitosan was used for modification with the amine group. Concentration and the numbers of amino groups present on the nanoparticle surface was as reported Bhattacharya et al., 3.25 [36], compared to our result, which similarly available amino group reached a value of 2.48 mmol/g.

## 5. Conclusions

This work reports on the synthesis and characterization of metal (γ-Fe_2_O_3_) oxide nanoparticles, which were further functionalized with chitosan using three different synthesis methods. HRP and ChOx were immobilized on chitosan functionalized metal oxide nanoparticles, and we found that the nanoparticles made using the suspension cross-linking process (MC2) proved to be the most suitable for obtaining the highest activity of immobilized enzyme, and that chitosan functionalized metal oxide nanoparticles made using covalent binding (MC3) are the most suitable for industrial applications. To summarize, the immobilization method of ChOx described here has an advantage as it allows for a wide range of biotechnology products with applications in diagnostics, bioaffinity chromatography and biosensors; this advantage is further enhanced by its magnetic properties. The synthesis of metal (γ-Fe_2_O_3_) oxide nanoparticles which were additionally functionalized with chitosan was successfully performed and their characterization was comprehensively described. Core-shell nanostructure, in which one inorganic nanomaterial is uniformly grown around a compositionally different nanocrystal core, produces a carrier for the deposition of a surface coating, which is then suitable for the attachment of organic molecules [74,75]. This technique is relatively simple, widely studied, and used in various scientific disciplines.

The results of the immobilization efficiency experiments were most successful with the use of PEHA, where high activity of the immobilized enzyme ChOx, up to 79%, was achieved. After immobilization of ChOx, the operational stabilities under repeated use of all three differently prepared supports (MC1, MC2 and MC3) were studied. We found that the residual ChOx activity on MC3 activated with PEHA after 5 successive cycles was 50% of the initial activity.

This study demonstrates the feasibility of these approaches and shows the competitiveness of enzyme immobilization on chitosan functionalized metal oxide micro- and nanoparticles. 

In general, taking into account environmental impacts and the current demands of the world’s biotechnological industries, immobilization of enzymes offers a reduction in production costs, enhancement in enzyme productivity, and the development of new techniques for increasing their shelf life.

## Figures and Tables

**Table 1:** Comparison of operational conditions for all three synthesis processes of chitosan functionalized metal oxide micro- and nanoparticles.

Parameters/Method	Microemulsion Process (MC1)	Suspension Cross-Linking Process (MC2)	Covalent Binding (MC3)
*Process Temperature*	40 °C, then 70 °C	room temperature	room temperature
*Stirring*	Ultrasonic (30 min), mechanical (120 min)	Ultrasonic (30 min), mechanical (4 h)	Ultrasonic (60 min), mechanical (12 h)
*Size distribution*	5–350 μm	10–200 μm	50–100 nm
*Mean diameter*	68.5 μm	44.2 μm	58.8 nm
*Saturation magnetization (Ms)*	4.0 emu/g	44.1 emu/g	14.2 emu/g

**Table 2:** Characterization properties comparison of chitosan functionalized metal oxide nanoparticles.

Properties	MC2	MC3
**Mean diameter**	44.2 µm	58.8 nm
**Saturation magnetization**	44.1 emu/g	14.2 emu/g
**Available amino groups**	0.02 mmol/g	2.48 mmol/g
**Immobilization efficiency**	35.0%	37.2%
**Residual activity**	79.0%	47.1%
**Reusability after 7 cycle**	0%	13%

## References

[1] Dhal J.P. (2015). Novel Metal Oxide Nanostructures for Adsorption and Photocatalytic Degradation of Organic Dyes from Aqueous Stream. Ph.D. Thesis.

[2] Patil K.C. (2008). Chemistry of Nanocrystalline Oxide Materials: Combustion Synthesis, Properties and Applications.

[3] Sulman E.M., Matveeva V.G., Bronstein L.M. (2019). Design of biocatalysts for efficient catalytic processes. Curr. Opin. Chem. Eng..

[4] Janardhanan S.K., Ramasamy I., Nair B.U. (2008). Synthesis of iron oxide nanoparticles using chitosan and starch templates. Transit. Met. Chem..

[5] Sahin S., Ozmen I. (2016). Determination of optimum conditions for glucose-6-phosphate dehydrogenase immobilization on chitosan-coated magnetic nanoparticles and its characterization. J. Mol. Catal. B Enzym..

[6] Naskar S., Sharma S., Kuotsu K. (2018). Chitosan-based nanoparticles: An overview of biomedical applications and its preparation. J. Drug Deliv. Sci. Technol..

[7] Vaseashta A.K., Mihailescu I.N. (2008). Functionalized Nanoscale Materials, Devices and Systems.

[8] Safdar R., Omar A.A., Arunagiri A., Iyyaswami R., Thanabalan M. (2018). Potential of Chitosan and its derivatives for controlled drug release applications—A review. J. Drug Deliv. Sci. Technol..

[9] Sonin D., Pochkaeva E., Zhuravskii S., Postnov V., Korolev D., Vasina L., Kostina D., Mukhametdinova D., Zelinskaya I., Skorik Y. (2020). Biological Safety and Biodistribution of Chitosan Nanoparticles. Nanomaterials.

[10] Osuna Y., Gregorio-Jauregui K.M., Gaona-Lozano J.G., de la Garza-Rodríguez I.M., Ilyna A., Barriga-Castro E.D., Saade H., López R.G. (2012). Chitosan-coated magnetic nanoparticles with low chitosan content prepared in one-step. J. Nano Mater..

[11] Samrot A.V., Shobana N., Durga Sruthi P., Sahithya C.S. (2018). Utilization of chitosan-coated superparamagnetic iron oxide nanoparticles for chromium removal. Appl. Water Sci..

[12] Laochai T., Mooltongchun M., Teepoo S. (2016). Design and Construction of Magnetic Nanoparticles Incorporated with a Chitosan and Poly (vinyl) Alcohol Cryogel and its Application for Immobilization of Horseradish Peroxidase. Energy Procedia.

[13] Gu T., Wang J., Xia H., Wang S., Yu X. (2014). Direct Electrochemistry and Electrocatalysis of Horseradish Peroxidase Immobilized in a DNA/Chitosan-Fe_3_O_4_ Magnetic Nanoparticle Bio-Complex Film. Materials.

[14] Lai G.-S., Zhang H.-L., Han D.-Y. (2009). Amperometric hydrogen peroxide biosensor based on the immobilization of horseradish peroxidase by carbon-coated iron nanoparticles in combination with chitosan and cross-linking of glutaraldehyde. Microchim. Acta.

[15] Tan X., Zhang J., Tan S., Zhao D., Huang Z., Mi Y., Huang Z. (2009). Amperometric Hydrogen Peroxide Biosensor Based on Horseradish Peroxidase Immobilized on Fe_3_O_4_/Chitosan Modified Glassy Carbon Electrode. Electroanalysis.

[16] Waifalkar P.P., Chougale A.D., Kollu P., Patil P.S., Patil P.B. (2018). Magnetic nanoparticle decorated graphene based electrochemical nanobiosensor for H2O2 sensing using HRP. Colloids Surf. B Biointerfaces.

[17] Ahmad S., Goswami P. (2014). Application of chitosan beads immobilized Rhodococcus sp. NCIM 2891 cholesterol oxidase for cholestenone production. Process Biochem..

[18] Tsai Y.-C., Chen S.-Y., Lee C.-A. (2008). Amperometric cholesterol biosensors based on carbon nanotube–chitosan–platinum–cholesterol oxidase nanobiocomposite. Sens. Actuators B Chem..

[19] Yapar E., Kayahan S.K., Bozkurt A., Toppare L. (2009). Immobilizing cholesterol oxidase in chitosan—Alginic acid network. Carbohydr. Polym..

[20] Kravanja G., Primožič M., Knez Ž., Leitgeb M. (2019). Chitosan-based (Nano) materials for Novel Biomedical Applications. Molecules.

[21] Alshabib M., Onaizi S.A. (2019). A review on phenolic wastewater remediation using homogeneous and heterogeneous enzymatic processes: Current status and potential challenges. Sep. Purif. Technol..

[22] Yu B., Cheng H., Zhuang W., Zhu C., Wu J., Niu H., Liu D., Chen Y., Ying H. (2019). Stability and repeatability improvement of horseradish peroxidase by immobilization on amino-functionalized bacterial cellulose. Process Biochem..

[23] Duarte Baumer J., Valério A., de Souza S.M.A.G.U., Erzinger G.S., Furigo A., de Souza A.A.U. (2018). Toxicity of enzymatically decolored textile dyes solution by horseradish peroxidase. J. Hazard. Mater..

[24] Hoang Thi T.T., Lee Y., Le Thi P., Park K.D. (2019). Engineered horseradish peroxidase-catalyzed hydrogels with high tissue adhesiveness for biomedical applications. J. Ind. Eng. Chem..

[25] Alapati K., Handanahal S.S. (2020). Characterization of cholesterol oxidase from a marine Streptomyces sp. and its cytotoxicity. Process Biochem..

[26] Arya S.K., Datta M., Malhotra B.D. (2008). Recent advances in cholesterol biosensor. Biosens. Bioelectron..

[27] Singh J., Srivastava M., Kalita P., Malhotra B.D. (2012). A novel ternary NiFe2O4/CuO/FeO-chitosan nanocomposite as a cholesterol biosensor. Process Biochem..

[28] Silva R.A., Carmona-Ribeiro A.M., Petri D.F.S. (2013). Enzymatic activity of cholesterol oxidase immobilized onto polymer nanoparticles mediated by Congo red. Colloids Surf. B Biointerfaces.

[29] Gopalan A.I., Lee K.-P., Ragupathy D. (2009). Development of a stable cholesterol biosensor based on multi-walled carbon nanotubes–gold nanoparticles composite covered with a layer of chitosan–room-temperature ionic liquid network. Biosens. Bioelectron..

[30] Solanki P.R., Kaushik A., Ansari A.A., Tiwari A., Malhotra B.D. (2009). Multi-walled carbon nanotubes/sol–gel-derived silica/chitosan nanobiocomposite for total cholesterol sensor. Sens. Actuators B Chem..

[31] Šulek F., Knez Ž., Habulin M. (2010). Immobilization of cholesterol oxidase to finely dispersed silica-coated maghemite nanoparticles based magnetic fluid. Appl. Surf. Sci..

[32] Križnik L., Vasić K., Knez Ž., Leitgeb M. (2018). Hyper-activation of ß-galactosidase from Aspergillus oryzae via immobilization onto amino-silane and chitosan magnetic maghemite nanoparticles. J. Clean. Prod..

[33] Podrepšek G.H., Knez Ž., Leitgeb M. (2013). Different preparation methods and characterization of magnetic maghemite coated with chitosan. J. Nano Part. Res..

[34] Zhu H.-Y., Jiang R., Xiao L., Li W. (2010). A novel magnetically separable gamma-Fe_2_O_3_/crosslinked chitosan adsorbent: Preparation, characterization and adsorption application for removal of hazardous azo dye. J. Hazard. Mater..

[35] Li G., Jiang Y., Huang K., Ding P., Chen J. (2008). Preparation and properties of magnetic Fe_3_O_4_-chitosan nanoparticles. J. Alloys Compd..

[36] Bhattacharya D., Sahu S., Banerjee I., Das M., Mishra D., Maiti T., Pramanik P. (2011). Synthesis, characterization, and in vitro biological evaluation of highly stable diversely functionalized superparamagnetic iron oxide nanoparticles. J. Nanopart. Res..

[37] Zemljič L.F., Tkavc T., Vesel A., Šauperl O. (2013). Chitosan coatings onto polyethylene terephthalate for the development of potential active packaging material. Appl. Surf. Sci..

[38] Bradford M.M. (1976). A rapid and sensitive method for the quantitation of microgram quantities of protein utilizing the principle of protein-dye binding. Anal. Biochem..

[39] Cao L. (2006). Carrier-Bound Immobilized Enzymes: Principles, Application and Design.

[40] Díaz-Hernández A., Gracida-Rodríguez J., García-Almendárez B., Regalado C., Núñez R., Amaro Reyes A. (2018). Characterization of Magnetic Nanoparticles Coated with Chitosan: A Potential Approach for Enzyme Immobilization. J. Nanomater..

[41] Shukla A., Gundampati R.K., Jagannadham M.V. (2017). Immobilization of Euphorbia tirucalli peroxidase onto chitosan-cobalt oxide magnetic nanoparticles and optimization using response surface methodology. Int. J. Biol. Macromol..

[42] López R., Pineda M., Hurtado G., León R., Fernández S., Saade H., Bueno D. (2013). Chitosan-Coated Magnetic Nanoparticles Prepared in One Step by Reverse Microemulsion Precipitation. Int. J. Mol. Sci..

[43] Reddy D.H.K., Lee S.-M. (2013). Application of magnetic chitosan composites for the removal of toxic metal and dyes from aqueous solutions. Adv. Colloid Interface Sci..

[44] Khmara I., Strbak O., Zavisova V., Koneracka M., Kubovcikova M., Antal I., Kavecansky V., Lucanska D., Dobrota D., Kopcansky P. (2019). Chitosan-stabilized iron oxide nanoparticles for magnetic resonance imaging. J. Magn. Magn. Mater..

[45] Fu C.-C., Tran H.N., Chen X.-H., Juang R.-S. (2020). Preparation of polyaminated Fe_3_O_4_@chitosan core-shell magnetic nanoparticles for efficient adsorption of phosphate in aqueous solutions. J. Ind. Eng. Chem..

[46] Gregorio-Jauregui K., Pineda M., Rivera-Salinas J., Hurtado G., Saade H., Martinez J., Ilyina A., Ul R., López R. (2013). One-Step Method for Preparation of Magnetic Nanoparticles Coated with Chitosan. J. Nanomater..

[47] Wang C., Yuan F., Pan J., Jiao S., Jin L., Cai H. (2014). A novel method for the determination of the degree of deacetylation of chitosan by coulometric titration. Int. J. Biol. Macromol..

[48] Kudr J., Haddad Y., Richtera L., Heger Z., Cernak M., Adam V., Zitka O. (2017). Magnetic Nanoparticles: From Design and Synthesis to Real World Applications. Nanomaterials.

[49] Gomes L., Paschoalin V., Mere Del Aguila E. (2017). Chitosan Nanoparticles: Production, Physicochemical Characteristics and Nutraceutical Applications. Rev. Virtual Quim..

[50] Mohammed M.A., Syeda J.T.M., Wasan K.M., Wasan E.K. (2017). An Overview of Chitosan Nanoparticles and Its Application in Non-Parenteral Drug Delivery. Pharmaceutics.

[51] Unsoy G., Yalcin S., Khodadust R., Gündüz G., Gunduz U. (2012). Synthesis optimization and characterization of chitosan-coated iron oxide nanoparticles produced for biomedical applications. J. Nano Part. Res..

[52] El-kharrag R., Halim S.S.A., Amin A., Greish Y.E. (2019). Synthesis and characterization of chitosan-coated magnetite nanoparticles using a modified wet method for drug delivery applications. Int. J. Polym. Mater. Polym. Biomater..

[53] Sahu S., Shera S.S., Banik R.M. (2019). Enhanced Reusability of Horseradish Peroxidase Immobilized onto Graphene Oxide/Magnetic Chitosan Beads for Cost Effective Cholesterol Oxidase Assay. Open Biotechnol. J..

[54] Cacicedo M.L., Manzo R.M., Municoy S., Bonazza H.L., Islan G.A., Desimone M., Bellino M., Mammarella E.J., Castro G.R., Singh R.S., Singhania R.R., Pandey A., Larroche C. (2019). Immobilized Enzymes and Their Applications. Biomass, Biofuels, Biochemicals: Advances in Enzyme Technology.

[55] Migneault I., Dartiguenave C., Bertrand M.J., Waldron K.C. (2004). Glutaraldehyde: Behavior in aqueous solution, reaction with proteins, and application to enzyme crosslinking. BioTechniques.

[56] Secundo F. (2013). Conformational changes of enzymes upon immobilisation. Chem. Soc. Rev..

[57] Luo L., Zhu L., Xu Y., Shen L., Wang X., Ding Y., Li Q., Deng D. (2011). Hydrogen peroxide biosensor based on horseradish peroxidase immobilized on chitosan-wrapped NiFe2O4 nanoparticles. Microchim. Acta.

[58] Bindhu L.V., Abraham E.T. (2003). Immobilization of horseradish peroxidase on chitosan for use in nonaqueous media. J. Appl. Polym. Sci..

[59] Cao X., Chen C., Yu H., Wang P. (2015). Horseradish peroxidase-encapsulated chitosan nanoparticles for enzyme-prodrug cancer therapy. Biotechnol. Lett..

[60] Mohamed S.A., Al-Malki A.L., Kumosani T.A., El-Shishtawy R.M. (2013). Horseradish peroxidase and chitosan: Activation, immobilization and comparative results. Int. J. Biol. Macromol..

[61] Amidi M., Mastrobattista E., Jiskoot W., Hennink W.E. (2010). Chitosan-based delivery systems for protein therapeutics and antigens. Adv. Drug Deliv. Rev..

[62] Labus K., Wolanin K., Radosiński Ł. (2020). Comparative Study on Enzyme Immobilization Using Natural Hydrogel Matrices—Experimental Studies Supported by Molecular Models Analysis. Catalysts.

[63] Mateo C., Torres R., Fernández-Lorente G., Ortiz C., Fuentes M., Hidalgo A., López-Gallego F., Abian O., Palomo J.M., Betancor L. (2003). Epoxy-amino groups: A new tool for improved immobilization of proteins by the epoxy method. Biomacromolecules.

[64] Shinya S., Fukamizo T. (2017). Interaction between chitosan and its related enzymes: A review. Int. J. Biol. Macromol..

[65] Faccio G. (2018). From Protein Features to Sensing Surfaces. Sensors.

[66] Fernandez-Lopez L., Pedrero S.G., Lopez-Carrobles N., Gorines B., Virgen-Ortíz J., Fernandez-Lafuente R. (2017). Effect of protein load on stability of immobilized enzymes. Enzym. Microb. Technol..

[67] Mohamad N.R., Marzuki N.H.C., Buang N.A., Huyop F., Wahab R.A. (2015). An overview of technologies for immobilization of enzymes and surface analysis techniques for immobilized enzymes. Biotechnol. Biotechnol. Equip..

[68] Huang J., Liu H., Zhang P., Zhang P., Li M., Ding L. (2015). Immobilization of cholesterol oxidase on magnetic fluorescent core-shell-structured nanoparticles. Mater. Sci. Eng. C Mater. Biol. Appl..

[69] Ghosh S., Ahmad R., Gautam V.K., Khare S.K. (2018). Cholesterol-oxidase-magnetic nanobioconjugates for the production of 4-cholesten-3-one and 4-cholesten-3, 7-dione. Bioresour. Technol..

[70] Guzik U., Hupert-Kocurek K., Wojcieszyńska D. (2014). Immobilization as a Strategy for Improving Enzyme Properties-Application to Oxidoreductases. Molecules.

[71] Gracida J., Arredondo-Ochoa T., García-Almendárez B.E., Escamilla-García M., Shirai K., Regalado C., Amaro-Reyes A. (2019). Improved Thermal and Reusability Properties of Xylanase by Genipin Cross-Linking to Magnetic Chitosan Particles. Appl. Biochem. Biotechnol..

[72] Bharathi D., Ranjithkumar R., Vasantharaj S., Chandarshekar B., Bhuvaneshwari V. (2019). Synthesis and characterization of chitosan/iron oxide nanocomposite for biomedical applications. Int. J. Biol. Macromol..

[73] Maldonado-Camargo L., Unni M., Rinaldi C. (2017). Magnetic Characterization of Iron Oxide Nanoparticles for Biomedical Applications. Methods Mol. Biol..

[74] Kong X. (2012). Simultaneous determination of degree of deacetylation, degree of substitution and distribution fraction of –COONa in carboxymethyl chitosan by potentiometric titration. Carbohydr. Polym..

[75] Dos Santos Z.M., Caroni A.L.P.F., Pereira M.R., da Silva D.R., Fonseca J.L.C. (2009). Determination of deacetylation degree of chitosan: A comparison between conductometric titration and CHN elemental analysis. Carbohydr. Res..

